# Effect of Regular Exercise and Functional Beverages on Changes in Body Weight and Waist Circumference in Healthy Japanese Subjects

**DOI:** 10.3390/medicina54040064

**Published:** 2018-09-04

**Authors:** Kazuki Ide, Masato Takeuchi, Tomotsugu Seki, Koji Kawakami

**Affiliations:** 1 Department of Pharmacoepidemiology, Graduate School of Medicine and Public Health, Kyoto University, Kyoto 606-8501, Japan; ide.kazuki.2r@kyoto-u.ac.jp (K.I.); takeuchi.masato.3c@kyoto-u.ac.jp (M.T.); seki.tomotsugu.54z@st.kyoto-u.ac.jp (T.S.); 2 Center for the Promotion of Interdisciplinary Education and Research, Kyoto University, Kyoto 606-8501, Japan

**Keywords:** food for specified health uses (FOSHU), body weight, body mass index, waist circumference, obesity

## Abstract

*Background and objectives*: The effects on anthropometry of several lipid-related foods for specified health uses (FOSHU) approved in Japan are not well known. We examined the effects of regular exercise and lipid-related FOSHU beverages on changes in body weight (BW) and waist circumference (WC) among factory and office workers. *Materials and Methods*: A total of 3002 subjects aged 20 years or older (2497 men and 505 women) received health check-ups in 2015 and 2016 and completed a lifestyle questionnaire. The association between regular exercise or lipid-related FOSHU beverage intake and changes in BW or WC were evaluated using trend test and linear regression analysis. *Results*: Regular exercise (≥1/week) was reported by 774 subjects (25.8%), and regular consumption of lipid-related FOSHU beverages (≥1/week) was reported by 1168 subjects (38.9%). The significant association between lipid-related FOSHU beverage intake and changes in BW was only observed among subjects with regular exercise (*p* for trend = 0.042). In the multivariable analysis, sex, older age, baseline weight, baseline body mass index (BMI), and regular exercise behavior were significantly associated with smaller changes in BW (*p* < 0.05). In WC, only the baseline values of WC and BMI were associated with one-year changes of WC. *Conclusions*: The combination of regular exercise and lipid-related FOSHU may be effective in maintaining BW.

## 1. Introduction

The prevalence of obesity and the number of overweight individuals is increasing worldwide, regardless of age and sex [1]. Global obesity is estimated to reach a prevalence rate of 18% in men and 21% in women by 2025, with both the global and regional mean body mass index (BMI) of adults increasing [2]. Occupation-related physical activity has decreased over the past 50 years, while the daily energy expenditure in the United States has dropped by more than 100 calories for men and women [3]. An increase in food energy supply and a decrease in physical activity are thought to be some of the drivers of the global obesity pandemic [4]. Therefore, the World Health Organization developed the Global Action Plan, which includes the voluntary global target to reduce the prevalence of insufficient physical activity and halt the rise in obesity [5]. Improvement in eating behavior and physical activity were set as targets both globally and regionally. The national movement to promote lifestyle improvements in Japan produced targets for reducing obesity and increasing regular exercise [6].

Foods for specified health uses (FOSHU) are food products that contain functional components which can have positive effects on health conditions or functions; declarations for specific health claims on food labels are approved for these foods [7]. FOSHU products are expected to improve dietary habits and contribute to health maintenance through the daily consumption of healthy food. Some FOSHU beverages are approved for health use and targeted toward individuals with high body fat or serum triglyceride levels. The ingredients in these FOSHU products are tea catechins, oolong tea polymerized polyphenols, coffee mannooligosaccharides, resistant maltodextrin, and quercetin glucosides. FOSHU products help suppress elevated serum triglyceride levels after meals, lower serum cholesterol or low-density lipoprotein cholesterol levels, or reduce body fat based on the findings of clinical studies using a small number of healthy subjects [8,9,10,11]. Although many of the lipid-related FOSHU beverages are not approved for making claims regarding changes in anthropometry, many clinical studies with short-term interventions have reported the association between FOSHU beverage intakes and changes in body weight (BW) or abdominal obesity with tea catechins [12,13,14,15,16], oolong tea [17], mannooligosaccharides [18,19,20,21], and quercetin [22,23,24]. However, the long-term effects of habitual consumption of FOSHU beverages on anthropometric data have not been adequately studied. Therefore, we aimed to evaluate the effect of lipid-related FOSHU beverage consumption and regular exercise on changes in BW and waist circumference (WC) using health check-up data of a Japanese cohort of factory and office workers.

## 2. Materials and Methods

### 2.1. Study Subjects

The cohort used in this study included a total of 7481 factory and office workers in Japan. Of these, 3962 completed a questionnaire of their medical history and lifestyle (response rate: 53.0%). Potential study subjects who were excluded included 296 subjects without annual health check-ups in 2015 and/or 2016, 139 who did not provide consent, 396 who were on medications, 170 who were pregnant, and 23 subjects who were involved in this study; therefore, the final study cohort consisted of 3002 subjects. The self-reported questionnaire included information on past medication, past medical history, past pregnancy and delivery, frequency of regular exercise (30 min or longer: “*How often do you exercise for 30 minutes or longer?*”), and number of lipid-related FOSHU beverages consumed (in units of bottles: “*How often do you take lipid-related FOSHU beverages?*”).

### 2.2. Data Collection

Body weight was measured while participants wore light clothing. Body height, weight, and WC were measured by a trained nurse at the health check-up. BW, BMI, WC, frequency of regular exercise, and number of FOSHU beverage bottles consumed were evaluated according to sex and age group. The reported volume of bottled FOSHU beverages consumed in the questionnaire ranged from 200–2000 mL. The suggested dose for the majority of the FOSHU beverages was either one bottle, or 200–500 mL with a meal. Hence, the total number of FOSHU beverage bottles consumed corresponded to the number of 200–500 mL bottles, depending on the volume of the bottle for each product. This study was conducted according to the Declaration of Helsinki, and the ethics committee of Kyoto University approved the use of these data and the study protocol (approval number: R1069). This study was conducted in accordance with the Ethical Guidelines for Medical and Health Research Involving Human Subjects in Japan.

### 2.3. Statistical Methods

Categorical variables were expressed as number and percentage. Continuous variables were summarized as mean (standard deviation) or median (interquartile range, IQR) according to the distribution of the data. The baseline BW, BMI, WC, frequency of regular exercise, and the number of lipid-related FOSHU beverages consumed were reported according to sex and age groups. Changes in BW and WC were calculated by subtracting the 2015 values from those of 2016, and the mean and standard error were presented. The *p* for trend was calculated using the Jonckheere-Terpstra test for ordinal variables, and the Cochran–Armitage trend test was used for categorical variables. The associations between beverage intake and changes in WC and BW were assessed separately among those who reported exercising at least once per week (*n* = 774) and those who reported no regular exercise (*n* = 2227). Sex-adjusted, age-adjusted, and multivariable-adjusted linear regression analyses were performed to examine the association among changes in BW, BMI, WC, and baseline factors. Multivariable-adjustment was performed for sex, age, baseline BW, BMI, or WC, frequency of regular exercise, and lipid-related FOSHU beverage intake. Two-sided tests were performed with a significance level of *p* < 0.05. Stata version 14.2 (STATA Corp., College Station, TX, USA) and SAS version 9.4 (SAS Institute Inc., Cary, NC, USA) were used for the statistical analysis.

## 3. Results

### 3.1. Subjects

Baseline characteristics of the study subjects are reported in Table 1. The WC increased with age among both men and women. Regular exercise was reported by 67.6% of men and 54.5% of women. Regular consumption of lipid-related FOSHU beverages (≥1/week) was reported by 40.2% of men and 32.5% of women. The ingredients of the lipid-related FOSHU beverages were quercetin glucosides (*n* = 1102), oolong tea polymerized polyphenols (*n* = 461), resistant maltodextrin (*n* = 107), tea catechins (*n* = 14), coffee bean mannooligosaccharides (*n* = 5), and others (*n* = 17).

### 3.2. Changes in Weight, BMI, and WC

Changes in BW and WC among men increased with age (all *p* for trend <0.001; Table 2), but no associations were observed among women (*p* for trend= 0.753, and 0.982, respectively). Changes in BW decreased with greater frequency of regular exercise in both men and women (*p* for trend = 0.012, and 0.044 respectively; Table 3).

### 3.3. Lipid-Related FOSHU Beverage Intake

Table 4 shows the association between lipid-related FOSHU beverage intake and changes in BW and WC. Changes in BW and WC were not associated with the frequency of lipid-related FOSHU beverage intake (*p* for trend = 0.731, and 0.269, respectively). The significant association between lipid-related FOSHU beverage intake and changes in BW was only observed among subjects with regular exercise (*p* for trend = 0.042; Table 5).

### 3.4. Multivariable-Adjusted Regression

Sex-adjusted, age-adjusted, and multivariable-adjusted regression analyses between baseline factors and changes in BW and WC are shown in Table 6. Older age and regular exercise behavior were associated with smaller changes in BW in the sex- and age-adjusted model. The baseline WC was also significantly associated with changes in WC. In the multivariable analysis, sex, older age, baseline weight, baseline BMI, and regular exercise behavior were significantly associated with smaller changes in BW. In WC, only the baseline values of WC and BMI were associated with one-year changes of WC.

## 4. Discussion

Using the health check-up data of Japanese factory and office workers, we examined associations between frequency of regular exercise and/or lipid-related FOSHU beverage intake with changes in BW and WC. We found a significant association between a greater frequency of lipid-related FOSHU beverages consumed with smaller changes in BW in subjects with regular exercise, but we did not observe this trend in those without regular exercise. This is the first study to demonstrate an association between lipid-related FOSHU beverage consumption and changes in BW and WC according to exercise frequency.

The observation period of this study was 12 months, which is much longer than most clinical studies evaluating the anti-obesity effect of lipid-related FOSHU beverages and their ingredients. Although some previous studies have used relatively longer observation periods, none were longer than 24 weeks. In previous studies, tea catechins were administered to healthy subjects for 12 [12,13,14,15,16] and 20 weeks [13]. BW, WC, abdominal fat, and visceral and subcutaneous fat have been shown to significantly decrease in high-dose catechin groups compared to low-dose or control groups [12,13,14,15]. Brown adipose tissue, which regulates non-shivering thermogenesis and adiposity, has been shown to increase significantly in the catechin group compared to the control group in healthy young women [16]. In another study, oolong tea polyphenols were given to healthy and overweight (25 kg/m^2^ ≤ BMI < 30 kg/m^2^) subjects for 12 weeks; abdominal fat area reduced significantly compared to the control group [17]. Similarly, coffee-derived mannooligosaccharides were given to healthy and overweight Japanese subjects for 12 weeks [18,19,20]. Abdominal, subcutaneous, and visceral fat areas significantly reduced compared to the control group [18,19]. However, in another study involving 60 overweight subjects from multiple ethnicities, total body volume, subcutaneous, and visceral adipose tissue significantly decreased compared to placebo in men, but not in women [20]. When indigestible dextrin was given to male subjects for 3 months, body fat decreased in all subjects, while visceral fat decreased in subjects with obesity [21]. Furthermore, when quercetin was administered to overweight and obese subjects for 12 [22,23] and 24 weeks [24], WC and abdominal, visceral, and subcutaneous fat areas were significantly reduced in the quercetin group compared to the placebo group.

The mechanisms of anti-obesity effects of the ingredients in lipid-related FOSHU beverages have been previously studied in basic and clinical studies. BW reduction mediated by the ingredients in tea are thought to be a result of decreased absorption of lipids and proteins, which reduces caloric intake and activates adenosine monophosphate (AMP)-activated protein kinase, which then suppresses adipogenesis and increases fatty acid oxidation [25]. The anti-obesity effects of oolong tea might be due to its inhibitory effect on pancreatic lipase activity [26]. Oolong tea polyphenol showed strong inhibition against pancreatic lipase, which leads to an inhibited absorption of lipids from the intestine and an increase in the excretion of these compounds [27]. Polyphenol-enriched oolong tea has been shown to increase fecal lipid excretion in healthy subjects [28]. Quercetin may show anti-obesity effects that are mediated by the attenuation of adipogenesis through the up-regulation of AMP-activated protein kinase (AMPK) and an increase in apoptosis of adipocytes through mitogen-activated protein kinase [29]. Quercetin reduces diet-induced liver fat accumulation by regulating lipogenesis in the transcription level [30]. Resistant maltodextrin suppresses lipid absorption and promotes the excretion of lipids into feces by delaying the release of fatty acid from the micelles, which is one of the first phases of the lipid absorption process [31]. However, the effects and mechanisms of lipid-related FOSHU beverage intake on long-term changes in BW or WC are unclear, and further studies are warranted to explain the long-term effects of these beverages.

In this study, greater frequencies of lipid-related FOSHU beverage intake combined with regular exercise were associated with smaller changes in BW; however, this association was not significant in the multivariable model. Baseline WC and regular exercise had a stronger effect on changes in WC compared to lipid-related FOSHU beverage consumption. Changes in WC were significantly associated with the frequency of lipid-related FOSHU beverage consumption and regular exercise. Mechanisms between the ingredients of lipid-related FOSHU beverages and regular exercise are not well studied. Voluntary exercise and green tea enhance the expression of genes related to energy utilization in mice fed a high-fat diet [32]. Therefore, lipid-related FOSHU beverage intake combined with regular exercise might be suggested for health benefits. Further studies are needed to examine the conditions under which the health benefits of lipid-related FOSHU beverages can be maximized.

The strength of this study is the relatively large number of subjects. However, there are also several limitations. First, information on food intake could not be collected. Second, information on the precise amount of lipid-related FOSHU beverages that were consumed could not be obtained, since our questionnaire was completed based on the number of lipid-related FOSHU beverages consumed without additional conditions. The recommended ingestion of lipid-related FOSHU beverages is 1–2 bottles per day. The highest lipid-related FOSHU beverage intake group (≥5 bottles/week) were presumed to be within the recommended range. Third, information regarding regular exercise and the amount of lipid-related FOSHU beverages were collected through a self-reported questionnaire; hence, misclassification could have occurred. This was an exploratory study and the questionnaire was not validated; therefore, additional studies with validated questionnaires are needed.

## 5. Conclusions

Changes in BW were smaller with greater frequencies of FOSHU intake with regular exercise behavior, but not among those without regular exercise behavior. The results of this study suggested that the combination of regular exercise and lipid-related FOSHU intake may be effective in maintaining BW.

## Figures and Tables

**Table 1:** Baseline characteristics of the study subjects according to sex and age groups.

	All	Age (years)				*p* for Trend
		20–29	30–39	40–49	≥50	
**Men**	**(*n* = 2497)**	**(*n* = 384)**	**(*n* = 952)**	**(*n* = 736)**	**(*n* = 425)**	
BW, kg	68.6 (62.4, 75.7)	66.4 (60.5, 73.4)	69.1 (69.1, 77.2)	69.7 (63.9, 76.2)	67.6 (61.8, 73.5)	0.373
WC, cm	83.0 (77.0, 89.0)	78.0 (74.0, 83.9)	82.5 (77.0, 89.0)	84.5 (79.0, 90.0)	84.0 (79.1, 90.0)	<0.001
Regular exercise frequency						
<1/month	783 (32.9)	95 (25.3)	294 (31.7)	270 (39.1)	124 (31.8)	0.036
1/month–1/week	1053 (44.2)	188 (50.1)	438 (47.3)	274 (39.7)	153 (39.2)	<0.001
2–4/week	489 (20.5)	80 (21.3)	179 (19.3)	136 (19.7)	94 (24.1)	0.651
≥5/week	58 (2.4)	12 (3.2)	16 (1.7)	11 (1.6)	19 (4.9)	0.208
Lipid-related FOSHU beverage intake, bottle/week						
<1	1493 (59.8)	251 (65.4)	544 (57.1)	421 (57.2)	277 (65.2)	0.830
1–2	438 (17.5)	70 (18.2)	196 (20.6)	119 (16.2)	53 (12.5)	0.002
3–4	304 (12.2)	37 (9.6)	119 (12.5)	99 (13.5)	49 (11.5)	0.384
≥5	262 (10.5)	26 (6.8)	93 (9.8)	97 (13.2)	46 (10.8)	0.012
**Women**	**(*n* = 505)**	**(*n* = 163)**	**(*n* = 118)**	**(*n* = 132)**	**(*n* = 92)**	
BW, kg	51.0 (46.5, 56.0)	50.7 (47.4, 54.4)	50.8 (45.5, 56.1)	52.4 (46.5, 57.2)	51.6 (46.1, 55.4)	0.641
WC, cm	73.5 (68.5, 79.5)	70.8 (66.5, 75.4)	73.0 (68.8, 78.5)	76.0 (71.0, 82.3)	76.7 (71.6, 85.2)	<0.001
Regular exercise frequency						
<1/months	210 (45.3)	60 (36.8)	48 (40.7)	60 (45.5)	42 (45.7)	0.103
1/month–1/week	181 (39.0)	65 (39.9)	48 (40.7)	43 (32.6)	25 (27.2)	0.026
2–4/week	72 (15.5)	31 (19.0)	16 (13.6)	14 (10.6)	11 (12.0)	0.057
≥5/week	1 (0.2)	1 (0.6)	0 (0.0)	0 (0.0)	0 (0.0)	0.505
Lipid-related FOSHU beverage intake, bottle/week						
<1	300 (59.4)	93 (57.1)	72 (61.0)	78 (59.1)	57 (62.0)	0.513
1–2	78 (15.5)	39 (23.9)	17 (14.4)	16 (12.1)	6 (6.5)	<0.001
3–4	49 (9.7)	18 (11.0)	15 (12.7)	11 (8.3)	5 (5.4)	0.118
≥5	37 (7.3)	7 (4.3)	8 (6.8)	12 (9.1)	10 (10.9)	0.037

BW, body weight; FOSHU, food for specified health uses; WC, waist circumference. Values are represented as median (IQR) for continuous variables and numbers (%) for categorical variables. SD, standard deviation. *p* for trend were calculated using the Jonckheere-Terpstra test for ordinal variables, and the Cochran–Armitage trend test was used for categorical variables.

**Table 2:** One-year changes in weight, and WC according to sex and age groups.

	Age (years)				*p* for Trend
	20–29	30–39	40–49	≥50	
**Men (*n* = 2497)**					
BW, kg/year	0.86 (0.15)	0.35 (0.08)	0.10 (0.09)	0.15 (0.10)	<0.001
WC, cm/year	1.09 (0.20)	0.19 (0.12)	−0.10 (0.15)	0.29 (0.21)	<0.001
**Women (*n* = 505)**					
BW, kg/year	0.31 (0.17)	0.13 (0.18)	0.72 (0.13)	0.03 (0.17)	0.753
WC, cm/year	0.86 (0.38)	−0.04 (0.40)	0.38 (0.43)	0.22 (0.60)	0.982

BW, body weight; WC, waist circumference. Values are represented as means (SE). SE, standard error. *p* for trend were calculated using the Jonckheere-Terpstra test.

**Table 3:** Changes in BW, and WC according to regular exercise frequency.

	Regular Exercise Frequencies				*p* for Trend
	<1/mo	1/mo–1/wk	≥5/wk	2–4/wk	
**Men (*n* = 2497)**					
BW, kg/year	0.43 (0.10)	0.37 (0.08)	0.24 (0.11)	−0.61 (0.33)	0.012
WC, cm/year	0.35 (0.12)	0.35 (0.10)	0.35 (0.15)	−0.79 (0.40)	0.192
**Women (*n* = 505)**					
BW, kg/year	0.63 (0.13)	0.19 (0.13)	−0.04 (0.28)	−1.1 (0.0)	0.044
WC, cm/year	0.66 (0.23)	0.66 (0.31)	−0.05 (0.45)	−6.5 (0.0)	0.555

BW, body weight; WC, waist circumference. Values are represented as means (SE). mo, month; SE, standard error; wk, week. *p* for trend were calculated using the Jonckheere-Terpstra test.

**Table 4:** Mean change in BW and WC according to lipid-related FOSHU beverage intake.

	Lipid-Related FOSHU Beverage Intake				*p* for Trend
	<1/wk	1–2/wk	3–4/wk	≥5/wk	
**All (*n* = 3002)**	(*n* = 1793)	(*n* = 516)	(*n* = 353)	(*n* = 299)	
BW, kg/year	0.35 (0.05)	0.44 (0.11)	0.29 (0.15)	0.01 (0.17)	0.731
WC, cm/year	0.35 (0.10)	0.34 (0.15)	0.37 (0.20)	−0.16 (0.20)	0.269

BW, body weight; WC, waist circumference. Values are represented as mean (standard error). *p* for trend were calculated using the Jonckheere-Terpstra test.

**Table 5:** Mean change in BW and WC according to the number of lipid-related FOSHU beverage intake with or without regular exercise.

	Lipid-Related FOSHU Beverage Intake				*p* for Trend
	<1/wk	1–2/wk	3–4/wk	≥5/wk	
**Regular exercise (+) (*n* = 774)**	(*n* = 439)	(*n* = 136)	(*n* = 79)	(*n* = 80)	
BW, kg/year	−0.05 (0.09)	0.53 (0.21)	0.67 (0.32)	−0.44 (0.34)	0.042
WC, cm/year	−0.18 (0.29)	0.64 (0.29)	0.69 (0.44)	−0.84 (0.43)	0.473
**Regular exercise (−) (*n* = 2227)**	(*n* = 1354)	(*n* = 380)	(*n* = 274)	(*n* = 219)	
BW, kg/year	0.48 (0.06)	0.41 (0.12)	0.19 (0.17)	0.17 (0.19)	0.067
WC, cm/year	0.53 (0.09)	0.24 (0.18)	0.28 (0.22)	0.09 (0.23)	0.075

BW, body weight; WC, waist circumference. Values are represented as mean (standard error). *p* for trend were calculated using the Jonckheere-Terpstra test.

**Table 6:** Regression analysis between baseline factors and changes in BW, body mass index, or WC.

	Sex- and Age-Adjusted *			Multivariable-Adjusted ^†^		
	β	(95% CI)	*p*-Value	β	(95% CI)	*p*-Value
Changes in BW, kg/year						
Men vs. women	0.10	(−0.11, 0.32)	0.340	0.36	(0.07, 0.64)	0.015
Age, years	−0.17	(−0.26, −0.09)	<0.001	−0.16	(−0.25, −0.08)	<0.001
Baseline BW, kg	0.00	(−0.01, 0.00)	0.157	−0.01	(−0.02, −0.01)	<0.001
Baseline body mass index, kg/m^2^	−0.01	(−0.02, 0.00)	0.016	−0.01	(−0.02, 0.00)	0.012
Regular exercise	−0.31	(−0.51, −0.11)	0.002	−0.20	(−0.39, −0.02)	0.032
Lipid-related FOSHU beverage intake	−0.07	(−0.15, 0.01)	0.097	0.01	(−0.06, 0.08)	0.760
Changes in WC, cm/year						
Men vs. women	0.20	(−0.16, 0.57)	0.277	1.21	(0.79, 1.63)	<0.001
Age, years	0.07	(−0.37, −0.08)	0.003	−0.01	(−0.17, 0.13)	0.823
Baseline WC, cm	−0.07	(−0.08, −0.06)	<0.001	−0.16	(−0.18, −0.14)	<0.001
Baseline body mass index, kg/m^2^	−0.02	(−0.03, −0.01)	<0.001	0.1	(0.08, 0.12)	<0.001
Regular exercise	−0.46	(−0.80, −0.12)	0.008	0.02	(−0.56, 0.06)	0.117
Lipid-related FOSHU beverage intake	−0.08	(−0.22, 0.07)	0.287	−0.04	(−0.20, 0.02)	0.116

* Sex was age-adjusted and age was sex-adjusted. ^†^ Adjusted for sex, age, baseline BW, body mass index, or WC, regular exercise, and lipid-related specified health beverage intake. BW, body weight; CI, confidence interval; FOSHU, food for specified health uses; WC, waist circumference.
